# Asymmetric dumbbell dimers simultaneously supporting quasi-bound states in continuum and anapole modes for terahertz biosensing

**DOI:** 10.1515/nanoph-2024-0254

**Published:** 2024-08-01

**Authors:** Jixin Feng, Xianghui Wang, Weinan Shi, Liang Ma, Yunyun Ji, Fei Fan, Shengjiang Chang

**Affiliations:** 12538 Institute of Modern Optics, Nankai University, Tianjin, China

**Keywords:** metasurface, bound states in continuum, multipole decomposition, biosensing

## Abstract

Multi-resonant metasurfaces are of great significance in the applications of multi-band nanophotonics. Here, we propose a novel metasurface design scheme for simultaneously supporting quasi-bound states in continuum (QBIC) and other resonant modes, in which QBIC resonance is generated by mirror or rotational symmetry breaking in oligomers while other resonant modes can be simultaneously excited by rationally designing the shapes of meta-atoms within oligomers. As an example, the simultaneous excitation of QBIC and anapole modes are demonstrated in a dimer metasurface composed of asymmetric dumbbell-shaped apertures. Based on the far-field multipole decomposition and near-field electromagnetic field distributions, the origin mechanisms of QBIC and anapole mode are elucidated. The symmetry breaking of dumbbell-shaped dimer results in QBIC. Within a certain asymmetric variation range, the contributions of toroidal dipole moment and electric dipole moment with approximately equal magnitudes remain dominant, which allows the anapole mode to always present. The effectiveness of the proposed design scheme is further confirmed by the experimental results identical with the evolutions of numerical simulation. In terahertz biosensing experiments, the anapole mode exhibits a higher sensitivity of 271.3 GHz (nmol/μl)^−1^, whereas the QBIC can achieve a lower detection limit of 0.015 nmol/μl and expands the detection range by almost an order of magnitude. Our findings are beneficial to designing multi-resonant metasurfaces with different resonance modes and promote the corresponding applications in the fields of biosensing, lasers, filtering, and nonlinearity.

## 1 Introduction

Metasurfaces are artificial electromagnetic microstructures composed of periodically arranged subwavelength structural units and exhibit powerful capabilities in controlling electro-magnetic field. Carefully designing the geometric structure and material properties of metasurface units could result in the excitation of different resonance modes including Fano resonance [1], [2], electromagnetic induced transparency (EIT) [3], dipole resonance [4], anapole [5–10], and quasi-bound states in continuum (QBIC) [11–19]. The strongly confined local fields formed in resonant metasurfaces can be used to enhance the interaction between light field and matters, which shows very strong application potential in the fields such as ultra-sensitive sensing. Wang et al. designed a QBIC resonator based on a metal split ring structure for ultra-sensitive terahertz (THz) sensing [20], with a sensing sensitivity as high as 674 GHz/RIU. To satisfy the requirements of multiband nanophotonic applications including frequency multiplex [21], [22] and multi-parameter sensing [23], [24], multi-resonant metasurfaces that can provide multiple local fields enhancements within a certain frequency band have gradually attracted considerable attentions. Hu et al. [25] designed an all-dielectric double resonance metasurface to achieve simultaneous detection of refractive index and temperature. In addition, multi-band resonances are highly likely to overlap with finger-print spectra of biological samples. Altug et al. [26] proposed a two-dimensional pixelated metasurface with a series of high-Q resonances at discrete frequencies to enable chemical identification and composition analysis of different analyte. The similar method was also applied in terahertz (THz) band by Lyu et al. [27]. Recently, different resonance modes have been demonstrated the capacity in enhancing distinct multiple sensing indicators. Shi et al. compared two different modes in sensing performance supported by a hybrid metal-dielectric metasurface [28]. Due to the difference in light field distributions, it is found that the dielectric-dominated QBIC mode performs a higher bulk sensitivity, whereas the mental-domainated QBIC exhibits a stronger surface affinity in the biotinstreptavidin bioassay. Therefore, simultaneously exciting multiple resonances with different modes is beneficial to bring about a comprehensive augmentation in the system’s overall performance including sensitivity, stability and detection range. However, although different mode resonant effects have been occasionally demonstrated in a few reports [29], there is still a lack of thorough research on the design scheme and origin mechanism.

Bound states in continuum (BIC) have gradually received a lot of attention in recent years. Theoretically, an ideal BIC exhibits an infinitely narrow linewidth resonance with an infinitely high quality-factor (Q-factor). Actually, due to limitations in structural size and material loss, BIC will degenerate into a quasi-BIC (QBIC) resonance with a limited Q-factor. Symmetry-protected QBIC resonance can be excited by introducing symmetry breaking (such as deformation, displacement, etc.) into symmetric structures. For example, Li et al. excited QBIC resonance by embedding eccentric holes in square silicon nanodisks [30]. Compared with single nanoparticles, the multi-particle arrangement of oligomers allows them to possess more adjustable structural parameters and higher degrees of freedom for manipulation. Oligomer structures can support complex mode collective behaviors due to electro-magnetic couplings between the constituent particles within the unit and between adjacent units. Therefore, introducing symmetry breaking into oligomers is a highly efficient way to generate QBIC resonance. Rivas et al. [31] and Abujetas et al. [32] both used metal rod dimers to excite QBIC resonance in the THz band. Ding et al. [33] and Hong et al. [34] observed QBIC resonances in square and circular tetramer structures, respectively. For oligomers, the shape variations of individual particles have become less necessary to induce QBIC [35], [36]. At present, the constituent particles of oligomers are often limited to simple regular geometric shapes, such as square [37], circle [38], rod-shaped [39], etc. If single structure supporting another resonance mode is utilized as the constituent particle within oligomers while symmetry breaking occurs between the constituent particles, it is highly possible to achieve simultaneous excitation of QBIC and other resonance modes.

The generation of anapole mode mainly originates from the interference cancellation in the far field between the dominant electric dipole (ED) moment and toroidal dipole (TD) moment. Metallic structures capable of generating anapole modes encompass a range of designs, including split-ring resonators [40], [41], dumbbell-shaped aperture structures, etc. Among them, the narrow gap in the middle of dumbbell-shaped structures can support an ED moment while the two circles at both ends can induce two oppositely rotating surface currents to form a TD moment. This special structural shape plays a pivotal role in exciting anapole mode. In 2013, Fedotov et al. first proposed a three-dimensional dumbbell-shaped metasurface supporting anapole mode [42]. Since then, various dumb-bell-based metasurfaces have been frequently designed to excite anapole resonances [43], [44]. In 2021, Li et al. [45] proposed a planar dumbbell-shaped aperture metasurface and found that the anapole mode can be reliably excited across various structural parameters.

In this work, a novel scheme based on distinct excitation conditions of QBIC and anapole modes is proposed to design an asymmetric dumbbell-shaped dimer (ADSD) metasurface for simultaneously generating different resonance modes. A comprehensive exploration encompassing numerical simulations, theoretical analysis, fabricated metasurfaces, experimental characterization, biosensing and other aspects of research are conducted. The results show that with introducing symmetry breaking, QBIC and anapole modes in different frequency bands can be simultaneously observed in the transmission spectrum of ADSD metasurface. The excitation of anapole mode presents a strong robustness over a certain asymmetric variation range. The biosensing experiments in THz time domain spectroscopy demonstrate the application potential of ADSD metasurfaces simultaneously supporting different resonance modes.

## 2 Designing ADSD metasurface for simultaneous excitation of QBIC and anapole

### 2.1 Structure design

**Figure 1:**
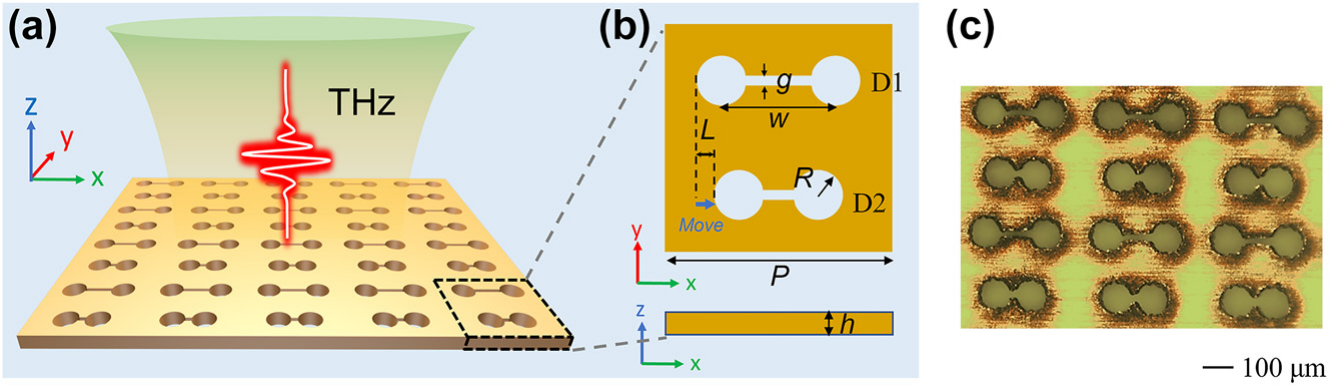
Structural design of ADSD metasurface. (a) Schematic diagram of ADSD metasurface. (b)Structural parameters of single unit cell. (c) Microscopic image of the fabricated metasurface.

As shown in Figure 1(a), the proposed ADSD metasurface is constructed from a copper (Cu) planar and consists of a periodic array of two dumbbells aperture resonators with different lengths. A *y*-linearly polarized THz wave is incident on the metasurface along the negative direction of *z* axis. Figure 1(b) gives the geometric structural parameters of single unit. For convenience of description, the two dumbbell-shaped apertures are respectively denoted as D1 and D2. The periodicity *P* along both the *x* and *y* directions take the value of 400 μm. The thickness *h* of the Cu planar is 100 μm. The air holes are designed with radius *R* = 48 μm and gap width *g* = 20 μm. The distance *w* between two holes of D1 is set to be 200 μm while that of the D2 is *w* − 2*L*. With increasing *L*, the unit structure exhibits asymmetry along the *x* direction, where the asymmetry parameter is defined as *α* = 2*L*/*w*. Figure 1(c) presents the microscopic image of the fabricated asymmetric metasurface when *α* = 0.4. The detailed fabrication information can be found in Section S2 of Supplementary Material. Oligomer structures have many advantages in exciting symmetry-protected QBIC resonance, and dimer structure is the simplest one. Additionally, the dumbbell-shaped aperture structure can support anapole mode. Therefore, the advantages of dimer and dumbbell-shaped structures are combined into the proposed ADSD structure. When *α* = 0, the special construction of the dumbbell-shaped structure modulates the electromagnetic field distribution within it to excite anapole mode. With increasing *α*, the symmetry breaking between dumbbell-shaped dimers enables the generation of QBIC resonance. Meanwhile, the excitation of anapole mode is still likely to be maintained.

### 2.2 Anapole mode induced by symmetric structure

**Figure 2:**
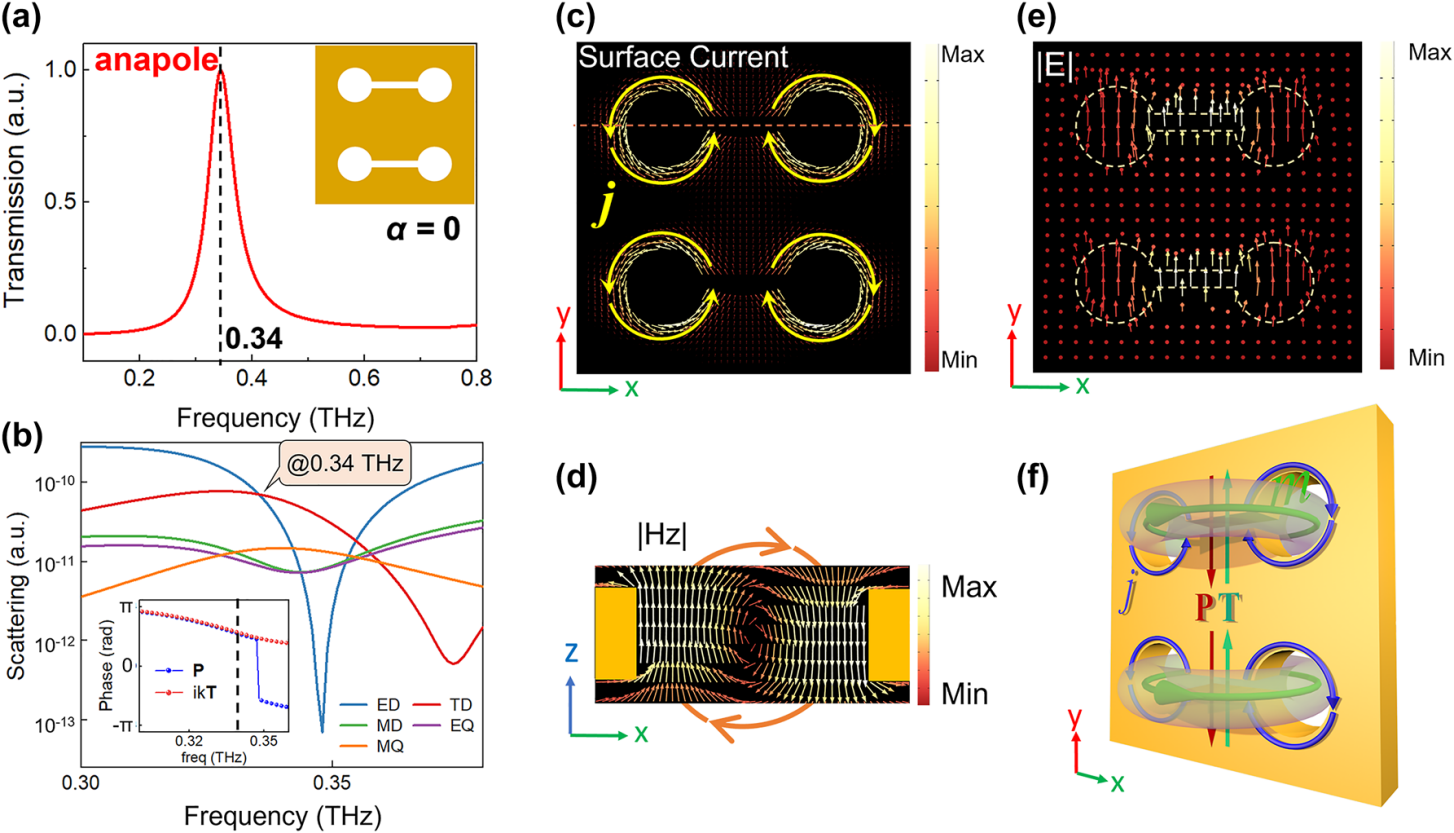
Anapole resonance of symmetric dumbbell dimer structure. (a) Simulated transmission spectrum of the dimer metasurface when *α* = 0. (b) Scattering power in the logarithmic scale of different multipoles calculated by multipolar decomposition. Insert: Phases of the ED moment **P** and TD moment *ik* **T**. The black dashed line is located at the resonance position in (a). Surface current (c) and electric field (e) in the *x*–*y* plane. (d) Magnetic field in the *x*–*z* plane along the red dotted line in (c). (f) Schematic diagram of the electromagnetic field distribution of anapole mode.

The far-field radiation pattern of ED moment **P** is identical with that of TD moment **T** [9]. When the dominant **P** and **T** satisfy the condition of interference cancellation in the far field, the anapole mode resonance will be excited. The dumbbell-shaped aperture can be used to enhance the contributions of **P** and **T**, which is beneficial to the generation of anapole resonance. Figure 2(a) shows the transmission spectrum of the symmetric dimer metasurface when *α* = 0. Due to the high reflectivity of metals to THz waves, the transmissions are nearly zero in most of band. However, a resonance peak at 0.34 THz occurs. The multipolar decomposition analyses can be used to compare the scattered powers of multipoles in the far field [41]. Figure 2(b) presents the multipolar contributions from various multipoles including ED, TD, magnetic dipole (MD), electric quadrupole (EQ) and magnetic quadrupole (MQ). At the resonance position, the magnitudes of ED and TD are nearly equal with each other and dominant compared to other multipoles. The far-field electric field radiated by the superposition of ED and TD can be expressed as **E**_tot_ ∼ (**P** − *ik* **T**) [41]. As shown in the insert of Figure 2(b), the condition of **P** = *ik* **T** are nearly satisfied at 0.34 THz, which means the interference cancellation will occurs in the far field and then an anapole resonance can be excited.

The near-field electromagnetic field distributions at 0.34 THz are further investigated, which could further confirm the existence of anapole mode. Figure 2(c) shows the surface current distribution in the *x*–*y* plane. Obviously, the D1 and D2 exhibit identical surface current patterns, due to the fact that the ADSD effectively behaves as a *P* × *P*/2 periodic unit when the asymmetry parameter *α* = 0. The surface currents encircle the dumbbell’s two holes and rotate in the opposing directions. Consequently, the currents around the left and right holes, respectively, generate the opposite magnetic fields along the +*z* and −*z* axes, which is confirmed in Figure 2(d). The electromagnetic patterns in Figure 2(c) and (d) clearly indicated the presence of TD moment **T** along +*y* axis. Additionally, Figure 2(e) reveals that there is also a contribution from an ED moment **P** aligned along the −*y* axis at 0.34 THz. The schematic diagram in Figure 2(f) illustrates the electromagnetic field distribution within the structure. The observed field distribution is coincided with multipole decomposition, providing evidence for the existence of non-radiative anapole mode. In addition, the results for different structural thickness (see Figure S2, Supplementary Material) show that the anapole mode is more easily excited in structures with higher *h*.

### 2.3 QBIC and anapole mode excited simultaneously in ADSD structures

**Figure 3:**
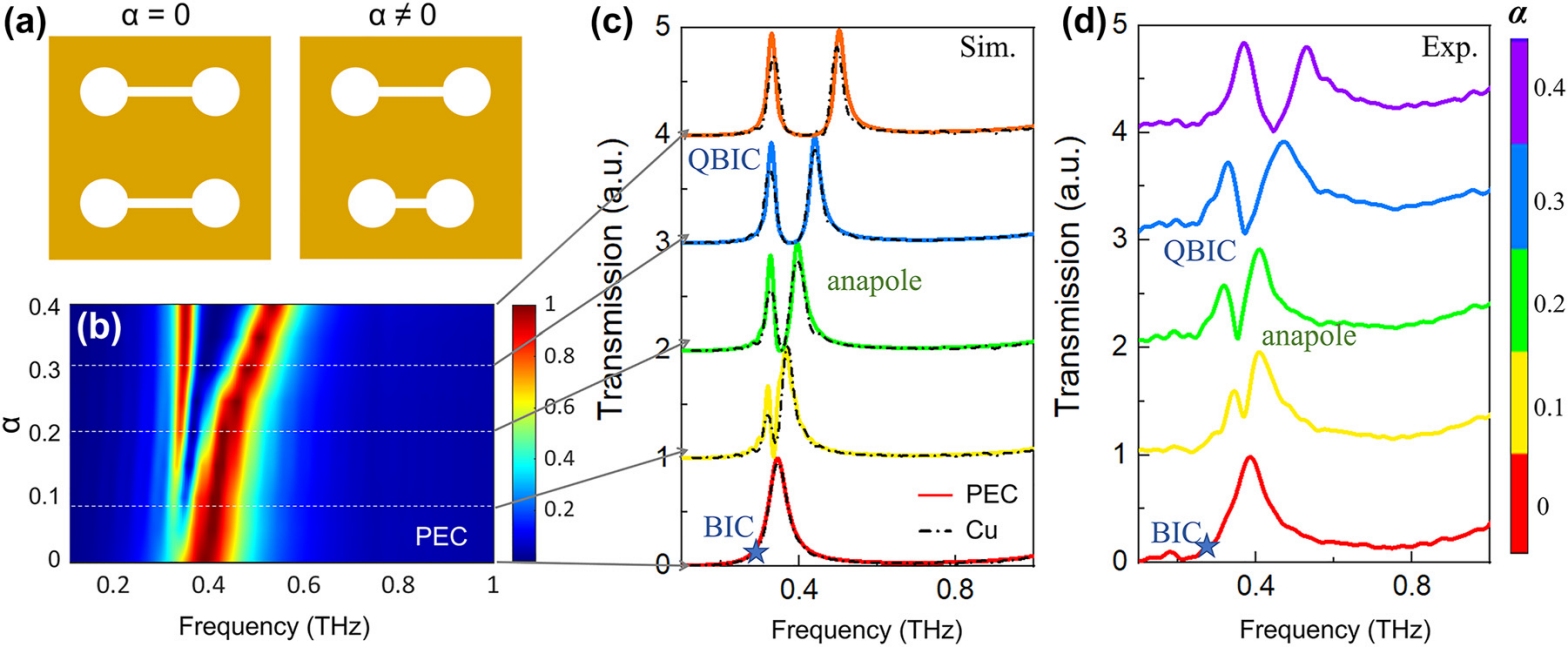
QBIC and anapole mode excited simultaneously in ADSD structure. (a) Schematic diagram of symmetric (*α* = 0) and asymmetric (*α* = 0.4) dumbbell dimers. (b) Transmission mapping versus frequency and *α*. Simulation (c) and experimental (d) results of transmission spectra when the asymmetry parameter *α* = 0, 0.1, 0.2, 0.3 and 0.4, respectively. In (c), the colored solid curves and black dashed lines respectively represent the results of PEC and Cu.

BIC mode can perfectly confine energy in a highly localized region, so theoretically BIC is an infinitely high Q-factor resonance. Fortunately, BIC can be degenerated into QBIC resonance with a finite *Q* factor by introducing symmetry breaking in structural unit. As shown in Figure 3(a), the symmetric dumbbell dimer evolves into asymmetric one by adjusting the asymmetry parameter *α*. Figure 3(b) shows the simulated transmission mapping as functions of frequency and *α*. When *α* = 0, only an anapole resonance is observed in the transmission spectrum. The dimer structure exhibits perfect symmetry, which implies that BIC is decoupled from the radiation channel and invisible in the transmission spectrum. As the value of *α* increases from 0 to 0.4, the symmetry broken within dimers allows BIC modes to leak into the radiation channel and then form a QBIC resonance. The linewidth of QBIC resonance broadens with increasing *α*. Simultaneously, the anapole resonance can always be observed in spectrum and shifts towards higher frequencies. Figure 3(c) and (d) present the simulated and experimental spectra for different values of *α*. In simulation, perfect electrical conductor (PEC) and Cu material are respectively adopted. The transmission spectra in Figure 3(c) indicate that both of resonance positions for those two materials are almost identical. As the symmetry broken is strengthened, the resonance position of QBIC nearly stabilizes at 0.32 THz, whereas the anapole mode undergoes a shift from 0.34 THz to 0.5 THz. The details about experimental spectroscopy characterization can be found in Section S3 of Supplementary Material. In the experimental spectra, the resonance peak of QBIC exhibits slight fluctuations centered around 0.35 THz, whereas the anapole mode shift from 0.39 THz to 0.53 THz. The discrepancies between the simulation and experimental results can be attributed to the unavoidable fabrication tolerances. Nevertheless, the overall evolution trends of the experimental spectra are basically consistent with those of the simulation results.

**Figure 4:**
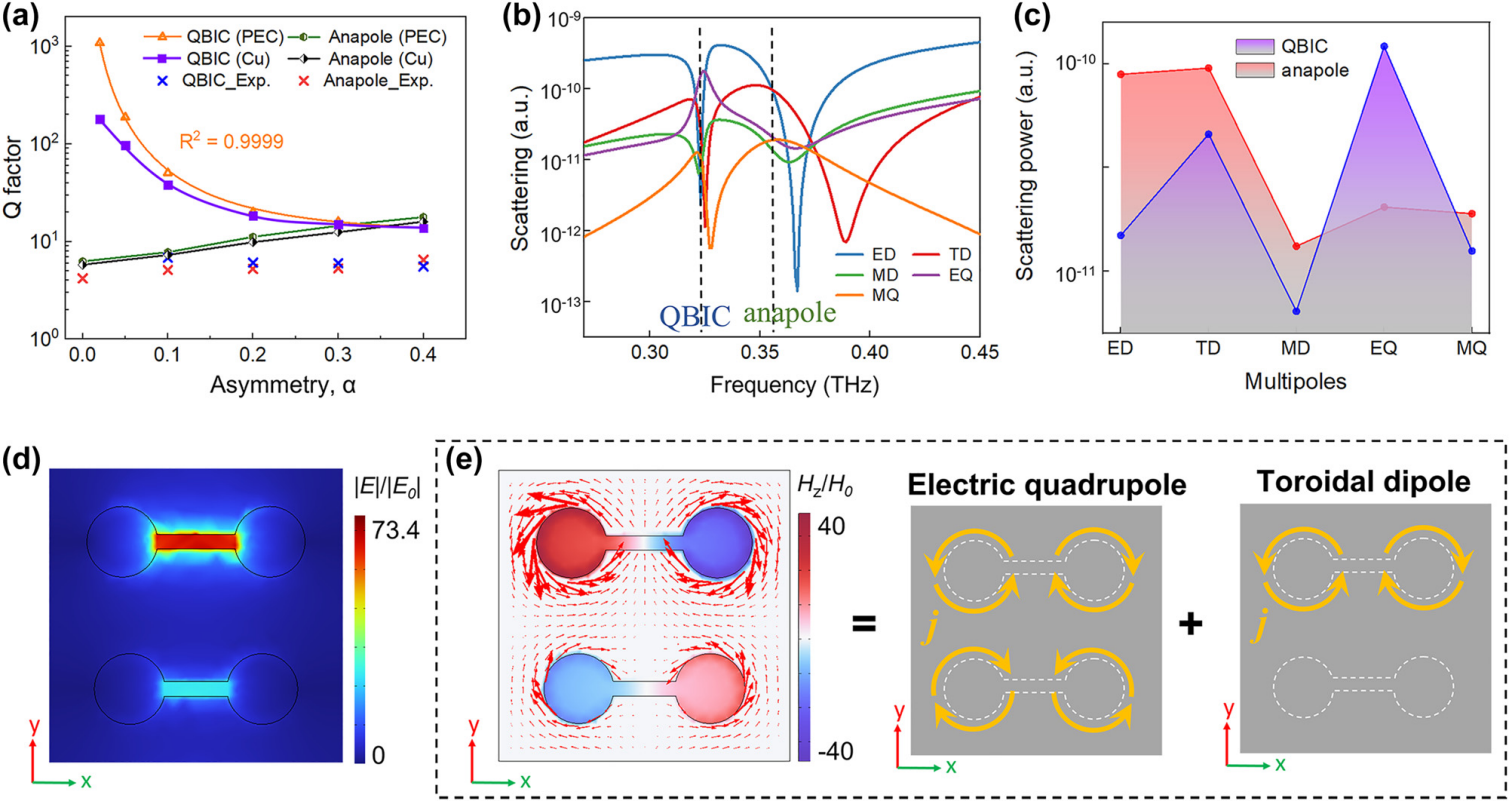
Investigations when structural symmetry is broken. (a) Variations of Q factors in the asymmetric logarithmic scale for QBIC mode and anapole mode with increasing α. (b) Scattering of different multipoles when α = 0.1. (c) Multipole contributions to QBIC mode and anapole mode respectively at 0.32 and 0.36 THz. For QBIC resonance at 0.32 THz, (d) electric field, (e) magnetic field component Hz and surface current induced by the superposition of EQ and TD. The surface currents are denoted by arrows.

For symmetrical-protected QBIC resonance, it is found that the *Q* factor and asymmetric parameters *α* should satisfy the relationship of *Q*∝1/*α* ^−2^. To calculate the *Q* factors, the Fano model is used to fit the resonances. The Fano model can be described as [11], [13].

(1) $$T=\left|a+ib+c/\left(\omega -\omega_{0}+i\gamma \right)\right|,$$

where *T* is the transmission and *ω*_0_ is the center frequency of the resonance peaks. *a*, *b* and *c* are the fitting parameters, respectively. *γ* is the damping rate. The *Q* factor can be expressed as *Q* = *ω*_0_/2*γ*. The changes in *Q* factors for both QBIC and anapole mode with increasing *α* are given in Figure 4(a). When the structure is constructed by PEC material, for QBIC mode, the determination coefficient of the fitting between *Q* factor and *α* is as impressively high as 0.9999, which clearly indicates that this mode is a symmetry-protected QBIC resonance. When the structure is set as Cu material, the *Q* factor is lower than that of PEC structure, due to material loss. Especially, the material loss induces a dramatic decline in the *Q* factor of QBIC resonance when *α* takes a smaller value. This phenomenon has also been studied in several previous studies [35], [36]. For anapole mode, the resonant *Q* factors of PEC and Cu material are nearly equivalent and a slight enhancement is observed with increasing *α*. The experimentally measured *Q* factors for both of anapole and QBIC modes are lower than the simulated results, which can be attributed to material loss, manufacturing tolerances and resolution of the characterization system. Nevertheless, the general trend coincides with the theoretical predictions. The simulation results in Figures 2, 3(c) and 4(a) and experimental results in Figure 3(d) firmly confirm that the ADSD structure can simultaneously support QBIC and anapole mode resonances. For the first time, the coexistence of those two different mode resonances is demonstrated in a single structure.

To further illustrate the electromagnetic mechanism of ADSD structure, the far-field analyses and near-field electromagnetic distributions are studied. Figure 4(b) shows the multipole decomposition results when *α* = 0.1 while Figure 4(c) gives the multipolar contributions to QBIC at 0.32 THz and anapole at 0.36 THz extracted from Figure 4(b). For anapole mode, when the structural symmetry is broken, ED and TD with approximately equal scattering magnitudes are still dominant. For QBIC mode, EQ provides the largest contribution while the contribution of TD is relatively weaker. The electric field of QBIC is mainly concentrated in the gap of D1 (shown in Figure 4(d)), while the magnetic field is dominantly distributed in the circle holes (shown in the left of Figure 4(e)). The surface current rotation directions of D1 and D2 are opposite. Additionally, the current density around D1 is much stronger than that of D2. The unbalanced surface current distribution of D1 and D2 can be partitioned into two components. One part is uniformly and reversely surrounding D1 and D2, which represents an EQ modal (shown in the middle of Figure 4(e)). Another part specifically surrounding D1 can be viewed as a TD model (shown in the right of Figure 4(e)). The electromagnetic field distribution and far-field analyses provide the conclusive evidence that the QBIC resonance is primarily a result of the interplay between EQ and TD.

### 2.4 Influence of structural asymmetry on anapole mode

**Figure 5:**
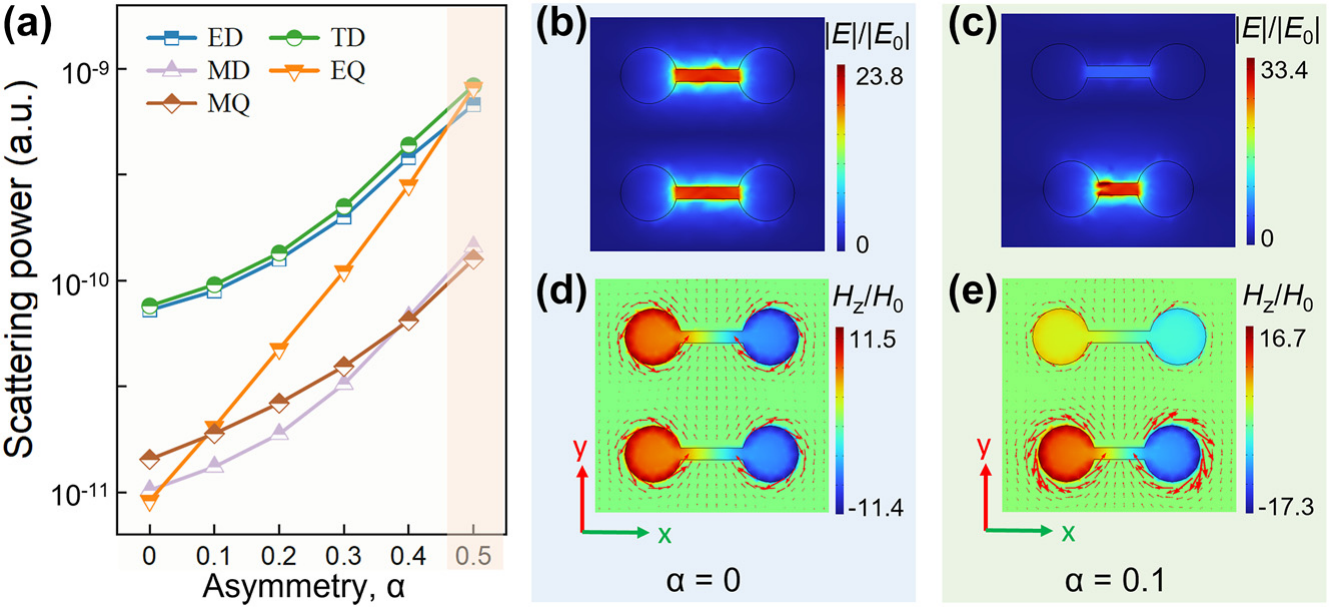
The influence of *α* on anapole mode. (a) Multipolar contributions to anapole mode for different *α* in the logarithmic scale. (b) Electric field and (d) magnetic component Hz at 0.34 THz in *xy*-plane when *α* = 0. (c) Electric field and (e) magnetic component Hz at 0.36 THz in *xy*-plane when *α* = 0.1.

Figure 5(a) shows the far-field contributions of multipoles to anapole mode with increasing *α*. As the symmetry broken is strengthened, the whole enhancement trend in the scattering magnitude can be observed for all multipoles, especially for EQ. The contributions from ED and TD remain dominant as the value of *α* varies from 0 to 0.4, which results that the anapole mode is always present. When the structure is symmetric (*α* = 0), the near-field electric field of anapole mode at 0.34 THz is uniformly distributed inside the gaps of D1 and D2 (see Figure 5(b)). When the symmetry breaking occurs (*α* > 0), the electric field is mainly concentrated within the gap of D2, which is different from that of QBIC mode (as shown in Figure 4(d)). According to Figure 5(b) and (c), it can be noted that the electric field enhancement is promoted with the increase of *α* (also see Figure S4 of Supplementary Material) and then results in the improvement of multipolar scattering magnitudes (as shown in Figure 5(a)). The magnetic component Hz and surface current density (red arrows) in Figure 5(d) and (e) are consistent with the multipole decomposition results in Figure 5(a). The far-field multipolar decomposition analyses and near-field electromagnetic distributions show that, within a certain asymmetric variation range (*α* < 0.4), in despite of the occurrence of symmetry breaking, the destructive interference between ED and TD is consistently maintained in D2, which endows the excitation of anapole mode with great robustness. However, it should be pointed out that the faster growing EQ becomes comparable to those two multipoles when *α* approaches 0.5 (see Figure 5(a)) and the anapole mode deteriorates into disappearance.

### 2.5 Sensing performance of ADSD metasurface

The vibrational and rotational energy levels of many biological macromolecules are located at the THz band [46]. Metasurfaces have been widely studied in the field of THz biosensing, due to the fact that their localized field enhancements are beneficial to strengthen the interactions between electromagnetic waves and samples. The sensing performance of ADSD supporting multi-mode resonances is investigated in this Section.

**Figure 6:**
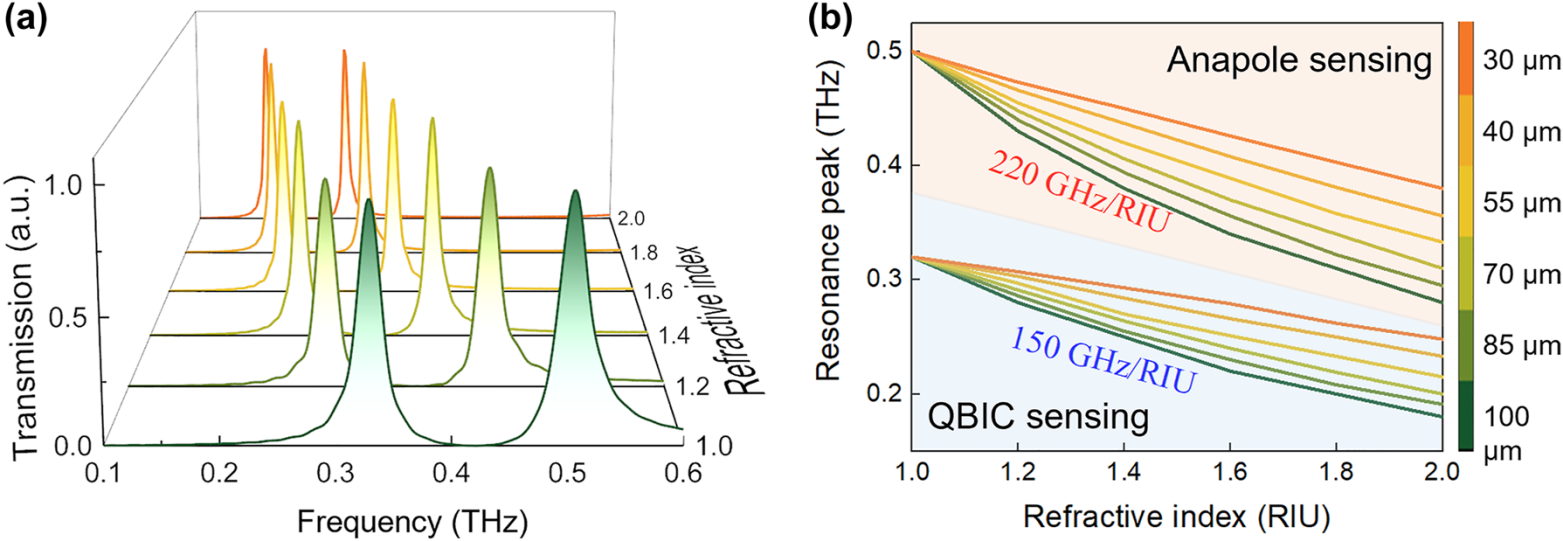
Simulation of RI sensing of anapole and QBIC modes. (a) Transmission spectra when RI changes from 1.0 to 2.0. (b) Resonance peak shifts of anapole and QBIC mode caused by analytes with different thickness and RI.

For the structure with *α* = 0.4, the refractive index (RI) sensing simulation is conducted by filling analytical media with varying thickness and RIs into D1 and D2. Figure 6(a) shows the transmission spectra as analyte RI varies from 1.0 to 2.0 when the thickness of sample is assumed to be 100 μm. As RI increases, both resonant peaks of QBIC and anapole mode shift to lower frequency. The changes in resonance peak position of anapole and QBIC mode versus the thickness and RI of analytes are shown in Figure 6(b). To evaluate the RI response, the sensing sensitivity is defined as *S* = Δ*f*/ΔRI. Both of the sensitivity of anapole and QBIC modes increase as the analyte thickness increases from 30 μm to 100 μm. When the analyte thickness is 100 μm, as RI increases from 1.0 to 2.0, the resonance peak of QBIC shifts from 0.33 THz to 0.18 THz, whereas the anapole resonance changes from 0.5 THz to 0.28 THz. The sensitivities of QBIC and anapole mode are 150 GHz/RIU and 220 GHz/RIU, respectively. The imaginary part of RI nearly has no impact on the sensing sensitivity (see Figure S3 in Supplementary Material).

**Figure 7:**
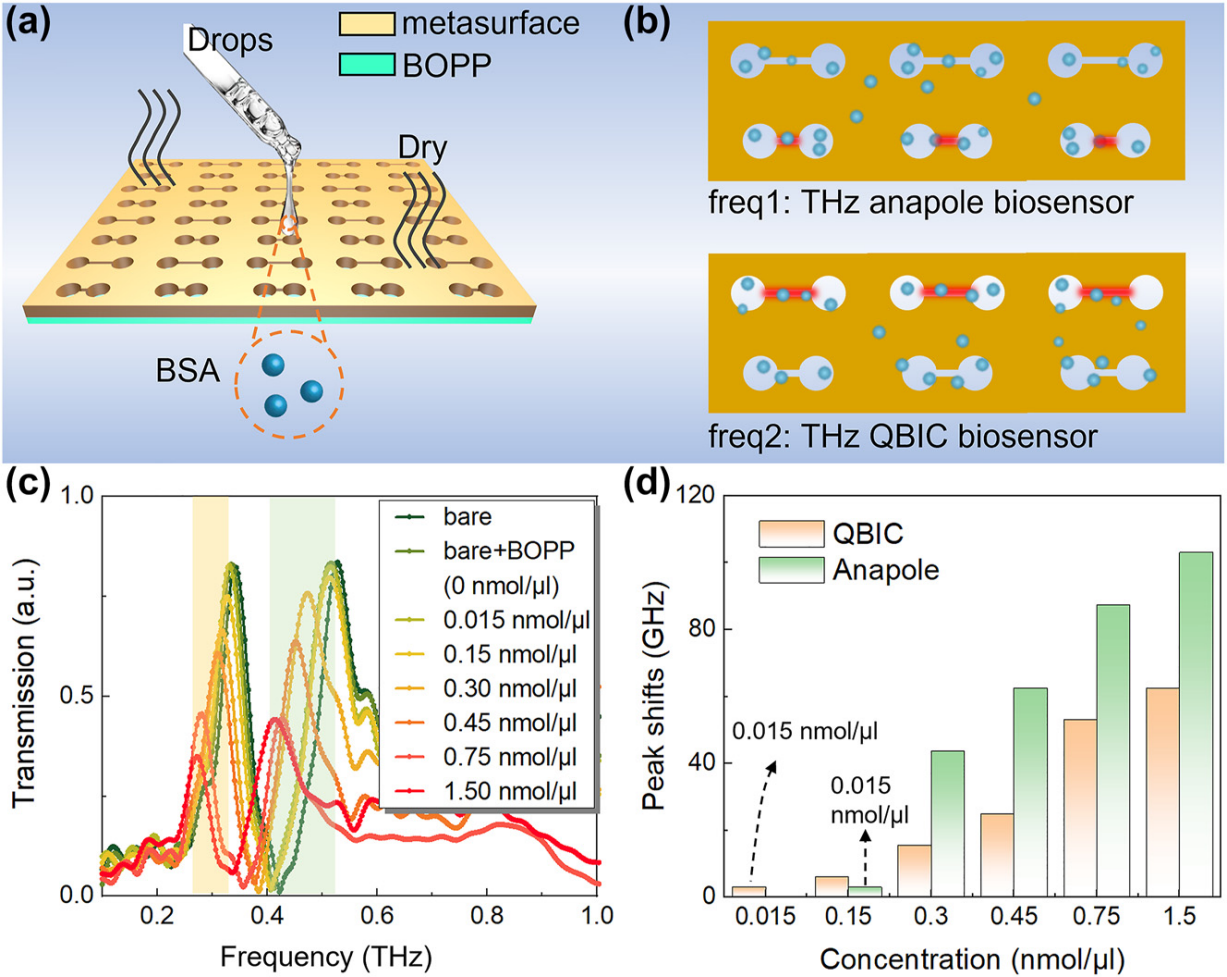
Experimental results of BSA biosensing. (a) Schematic diagram of sample processing. (b) Electric field interactions with BSA for anapole mode and QBIC. (c) Changes of transmission spectra for different BSA concentration. (d) Resonance peak shifts of QBIC and anapole mode.

Serum albumin, a crucial component in human plasma, plays a vital role in maintaining osmotic pressure. The blood albumin concentration is altered in numerous diseases including cirrhosis and nephrotic syndrome. Therefore, the detection of serum albumin concentration can provide indicators for auxiliary diagnosis and prognosis. Sensing performance of the proposed ADSD structure is experimentally conducted by taking the concentration detection of bovine serum albumin (BSA) as an example. Figure 7(a) shows sample processing for BSA concentration biosensing. It should be pointed out that ADSD is a hollow structure without a substrate. In order to provide a support for the analyte, a 40-μm-thick biaxially oriented polypropylene (BOPP) tape is attached to the structure back. More details about sample fabrication can be found in Section S2 of Supplementary Material. The interactions of BSA samples with QBIC and anapole mode are illustrated in Figure 7(b). The difference in the electric field distributions of those two modes implies that there is the discrepancy in light–matter interactions. Therefore, QBIC and anapole mode are likely to exhibit different sensing performances. As the BSA concentration varies from 0 nmol/μl to 1.5 nmol/μl, the experimentally measured transmission spectra are shown in Figure 7(c). It is worthily noted that with increasing BSA concentration, the peaks of both resonances decrease significantly, due to the stronger absorption in higher concentration of solution. Consistent with the simulation results (see Figure 6(a) and S3(b)), as the concentration increases, the two resonance peaks shift to lower frequency.

Figure 7(d) presents resonance peak shifts of QBIC and anapole modes for different concentrations. One of pivotal metrics for evaluating sensing performance is the sensitivity, which is usually used to quantify the optical response rate triggered by variations in sample concentration. As compared to QBIC, anapole mode is more sensitive over a concentration range from 0.3 nmol/μl to 1.5 nmol/μl. The maximum sensitivity of anapole mode reaches 271.3 GHz (nmol/μl)^−1^ in measurement, while that of QBIC is 93.6 GHz (nmol/μl)^−1^. In biosensing, a comprehensive evaluation of sensor performance necessitates the utilization of multiple indicators. Aside from the sensitivity, the detection limit (DL) is another key indicator for signifying the minimum identifiable concentration level [47], [48]. For example, in clinical, the diagnosis of kidney disease often relies on the detection of trace amounts of albumin in human urine [49]. According to Figure 7(d), it can be also found that when the concentration is reduced to 0.015 nmol/μl, the QBIC resonance still has a frequency shift of 3 GHz, while the frequency shift of anapole resonance cannot be observed. As compared with anapole mode, the DL of QBIC is lowered by almost an order of magnitude.

**Figure 8:**
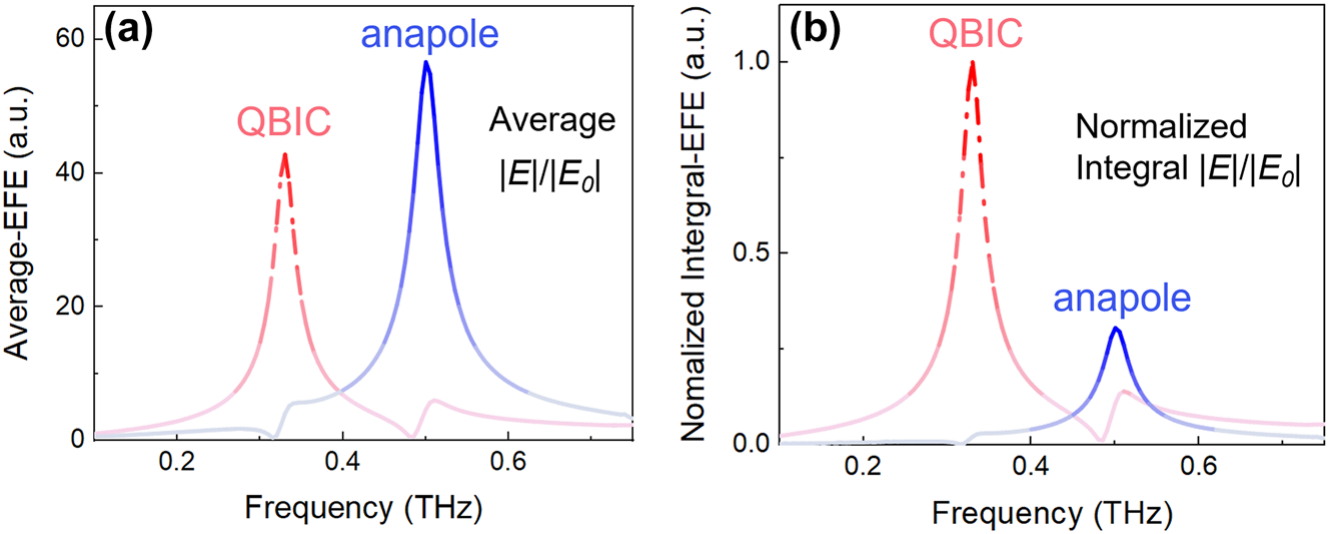
Average-EFE (a) and normalized Integral-EFE (b) for QBIC and anapole mode, respectively. Here, *α* = 0.4.

These distinct sensing performance in sensitivity and DL can be attributed to the difference in electric field distributions of those two modes. According to Figures S4(d) and 7(b), the hot spots of QBIC mode located within the gap in D1, whereas the hot spots of anapole mode is confined inside the gap in D2. For *α* = 0.4, the field enhancement of anapole mode is stronger than that of QBIC, whereas the field-enhanced region of QBIC is larger than that of anapole mode (see Figure S4 of Supplementary Material). To quantitatively evaluate the difference between those two aspects, the average electric field enhancement (Average-EFE) and integral electric field enhancement (Integral-EFE) are respectively defined as

$$\text{Average }-\text{ EFE }=\;\underset{V_{i}}{\int }\frac{\left|E\left(r\right)|/|E_{0}\right|}{V_{i}}dr,$$

$$\text{Integral }-\text{ EFE }=\;\underset{V_{i}}{\int }|E\left(r\right)|/|E_{0}|dr,$$

here, *V*_*i*_ is the volume within the gaps and the subscript *i* represents as 1 or 2. The results for QBIC and anapole mode are shown in Figure 8. Compared with QBIC mode, the Average-EFE of anapole mode is stronger (see Figure 8(a)), which means that the more strengthened interaction between THz wave and analytes can result in a higher sensitivity. However, according to Figure 8(b), the normalized Integral-EFE of QBIC is almost three times larger than that of anapole mode. Additionally, there is a wider hot spot for QBIC mode (see Figures S4(d)), which implies a larger sensing volume for accommodating analytes. The significantly spacious sensing region with field enhancement facilitates QBIC mode to effectively detect samples with much lower concentrations. The experimental results in sensitivity and DL of QBIC and anapole demonstrate the superiority of different resonant modes in improving the overall sensing performance of metasurfaces.

## 3 Conclusions

In summary, a novel metasurfaces designing scheme for simultaneously supporting QBIC and other resonant modes is proposed. QBIC resonance is generated by mirror or rotational symmetry broken in oligomers while other resonant modes can be simultaneously excited by rationally designing the shapes of meta-atoms within oligomers. An ADSD structure is taken as an example to simultaneously excite QBIC and anapole mode in THz band. Based on far-field multipole decomposition and near-field electromagnetic field distributions, the generation mechanisms of QBIC resonance and anapole resonance are revealed. The results also show that the excitation of anapole mode presents a strong robustness within a certain asymmetric variation range. The experimental results are identical with the evolutions of numerical simulation and further confirm the effectiveness of the proposed design scheme. In addition, the performance of ADSD structure in biosensing is also explored. The maximum sensitivities of anapole and QBIC are respectively 271.3 GHz (nmol/μl)^−1^ and 93.6 GHz (nmol/μl)^−1^, with DLs of 0.15 nmol/μl and 0.015 nmol/μl in experiment. The biosensing results shows the superiority of different mode resonant metasurface in improving the overall sensing performance. The proposed scheme can inspire the design of multi-resonant metasurfaces and further promote the applications of multi-band nano-photonics in sensing and nonlinear optics.

## Supplementary Material

Supplementary Material Details
