# Supplementary material for: Asymmetric dumbbell dimers simultaneously supporting quasi-bound states in continuum and anapole modes for terahertz biosensing
Source: Nanophotonics. 2024 Aug 1;13(21):4007–17. doi: 10.1515/nanoph-2024-0254 (PMC11501055; doi:10.1515/nanoph-2024-0254)
Supplement: Supplementary file 1 — Supplementary Material Details [file j_nanoph-2024-0254_suppl_001.docx]

**Supplementary material:**

Asymmetric dumbbell dimers simultaneously supporting quasi-bound states in continuum and anapole modes for terahertz biosensing

Jixin Feng^a^, Xianghui Wang^a*^, Weinan Shi^a^, Liang Ma^a^, Yunyun Ji ^a^, Fei Fan ^a*^, Shengjiang Chang ^a^

*^a^ Institute of Modern Optics, Nankai University, Tianjin Key Laboratory of Micro-scale Optical Information Science and Technology, Tianjin 300350, China*

*Corresponding author: [wangxianghui@nankai.edu.cn](mailto:wangxianghui@nankai.edu.cn); [fanfei@nankai.edu.cn](mailto:fanfei@nankai.edu.cn)

1. **Numerical Method**

In this study, the numerical calculations including transmission spectra, electromagnetic field distribution were performed with finite difference time domain method (Lumerical FDTD Solutions 2023 R1). Periodic boundary conditions were taken along the x and y directions, while perfect match layer (PML) boundary conditions were taken in the z directions. The y-polarized plane wave with a frequency ranging from 0.1 THz to1.0 THz is incident on the metasurface along the negative direction of z axis. The frequency points are set as 500. The conductivity of copper is set as 3×10^6^ S m^-1^.

1. **Fabrication**

Firstly, the surface of a 100 μm-thick Cu planar was thoroughly cleaned with alcohol and this process was repeated by multiple times. Then, the cleaned Cu planar was dried in an oven at 70°C for a duration of 30 minutes. Lastly, the ADSD structure was fabricated with the laser etching method. The etched area of each structure is 1 cm^2^ (25×25 units). In the fabrication of THz biosensing samples, a BOPP tape was attached on the back of ADSD structure. Then, 20 μl of BSA solutions with different concentrations were dropped onto to it. Lastly, the samples were placed in an environment of 60 °C for 20 minutes for drying.

1. **Terahertz-time domain spectroscopy characterization**

In order to demonstrate the spectral response of the ADSD structure, the experiment was conducted with a terahertz-time domain spectroscopy (THz-TDS) system as shown in Figure S1. A femtosecond laser with a central wavelength of 780 nm was irradiated onto a photoconductive antenna (PCA) to generate THz waves. When there was no sample placed in the light path, the transmitted electric field intensity was *E_ref_*(***t***). When the sample placed in the light path, the transmitted electric field intensity was *E_sam_*(***t***). After Fourier transform, their spectral information can be obtained respectively as *E_ref_*(***ω***) and *E_sam_*(***ω***). The transmission spectrum of the sample can be obtained by *T* =| *E_sam_*(***ω***)/ *E_ref_*(***ω***)|.


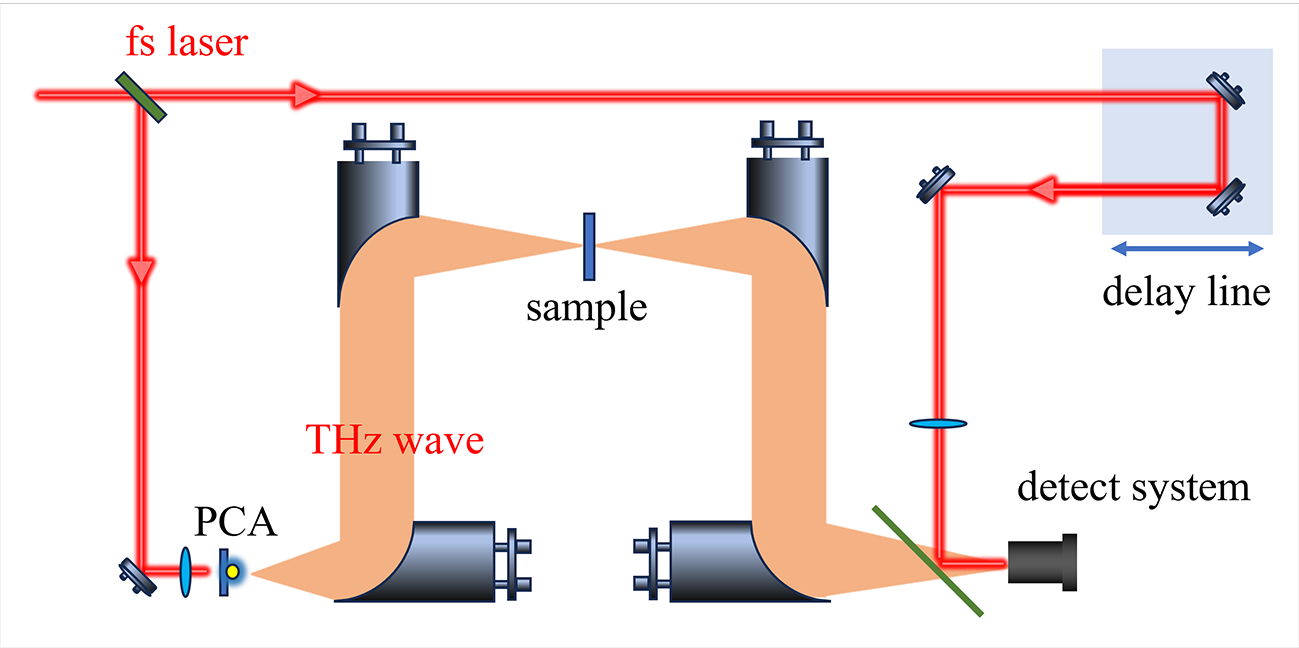


**Figure S1.** Schematic of THz-TDS system

**S4 The influence of structure thickness**


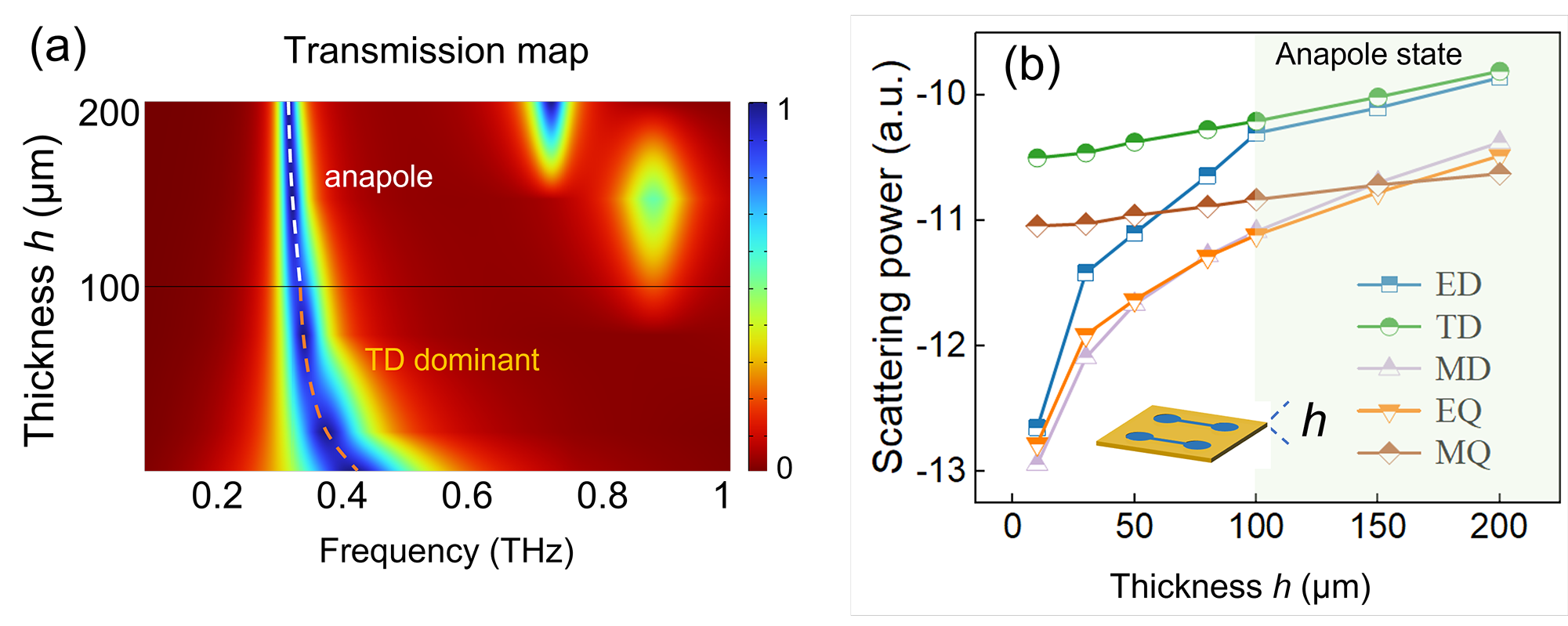


**Figure S2.** (a) Transmission mapping versus frequency and structure thickness *h* when *α* = 0. (b) Evolutions of multipolar contributions in the logarithmic scale for different *α*.

The transmission spectra for symmetrical structure (*α* = 0) with varying thickness *h* are shown in Figure S2(a). As *h* increases from 10 µm to 100 µm, a significant reduction in the resonance linewidth is observed and accompanied by a discernible shift towards lower frequencies. However, when *h* ranges from 100 µm to 200 µm, the changes in linewidth and resonance position become progressively less pronounced. Multipole decomposition analysis is conducted to explain the mechanism of this evolution. Figure S2(b) shows the multipolar contributions at the resonance positions for structures with different thickness. As *h* ranges from 0 µm to 100 µm, the contribution of TD always dominates. However, with the increase of *h*, the contributions of ED, MD, and EQ significantly increase, especially ED. Due to the disturbance of the three multipoles, the resonance position changes significantly. The interference destruction between ED and TD becomes more obvious and results in a decrease in the resonance line width. When *h* approaches 100 µm, the contribution of ED becomes comparable to that of TD. As *h* exceeds 100 µm, the increase of ED contributions slows down, and the contributions of ED and TD are approximately equal and dominant, which implies the excitation of anapole mode.

**S5 The influence of imaginary part of RI on sensing**


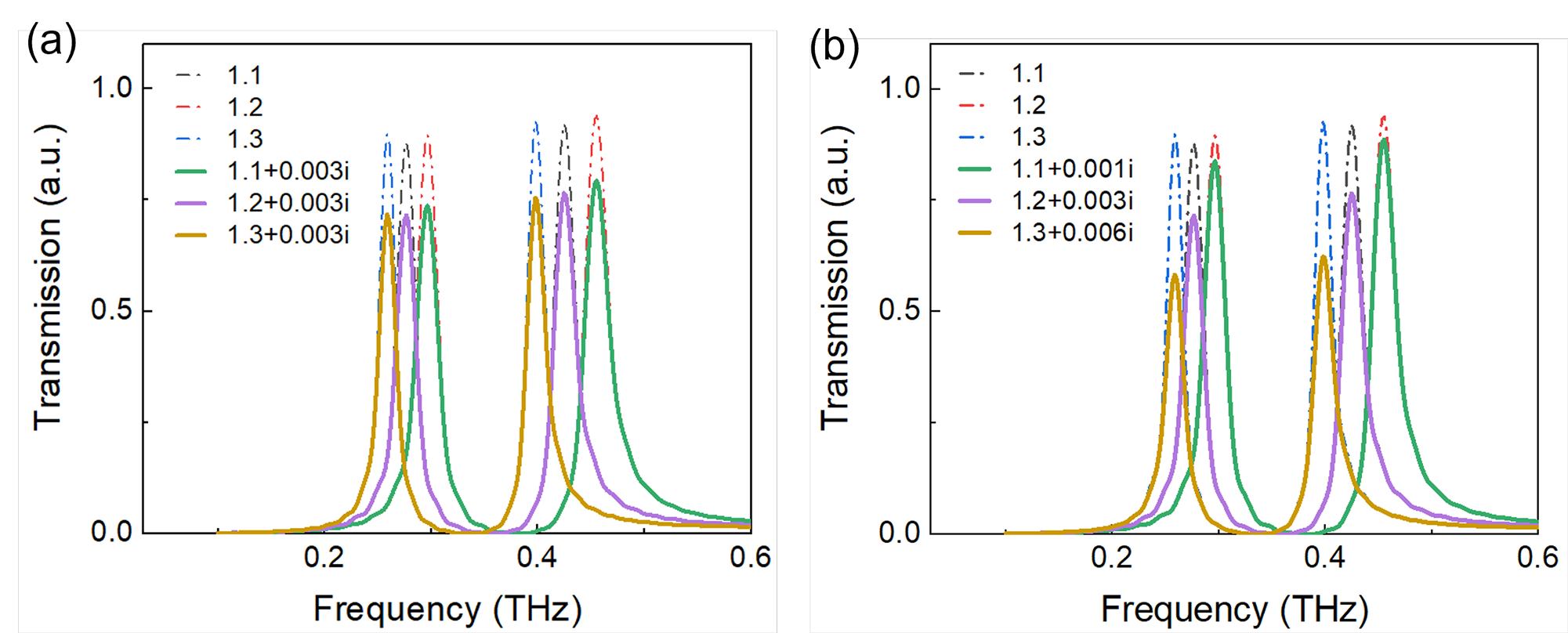


**Figure S3.** Simulation of RI sensing of anapole and QBIC modes for different imaginary part of RI. (a) Transmission spectra when *k* = 0 and *k* = 0.003, respectively. (b) Transmission spectra when *k* varies with the real part of RI.

To facilitate the discussion in the main text, the imaginary part of the refractive index is neglected in Figure 6. However, in practical applications, the optical losses caused by sample absorption and scattering are often inevitable. Taking into account optical losses, RI can be expressed as *n* = *n'* + *ik*, where *k* represents as the extinction coefficient for quantifying optical losses. The analyte with real part of RI varying from 1.1 to 1.3 is filled into D1 and D2 of the ADSD structure with *α* = 0.4. Figure S3(a) compares transmission spectra when *k* = 0.003 and *k* = 0, respectively. Compared with the case of *k* = 0, only the resonance intensities are weakened when *k* = 0.003, while the resonances positions of QBIC and anapole modes remain unchanged. The sensitivity is dependent on the shift of resonance position. Therefore, the sensing sensitivity of ADSD structure is unaffected by optical losses. Figure S3(b) further explores the results for different real part of RI with varying *k*. It also shows that as compared with the case of *k* = 0, the resonant peaks still maintain unchanged.

**S6 Near field electromagnetic field distribution**


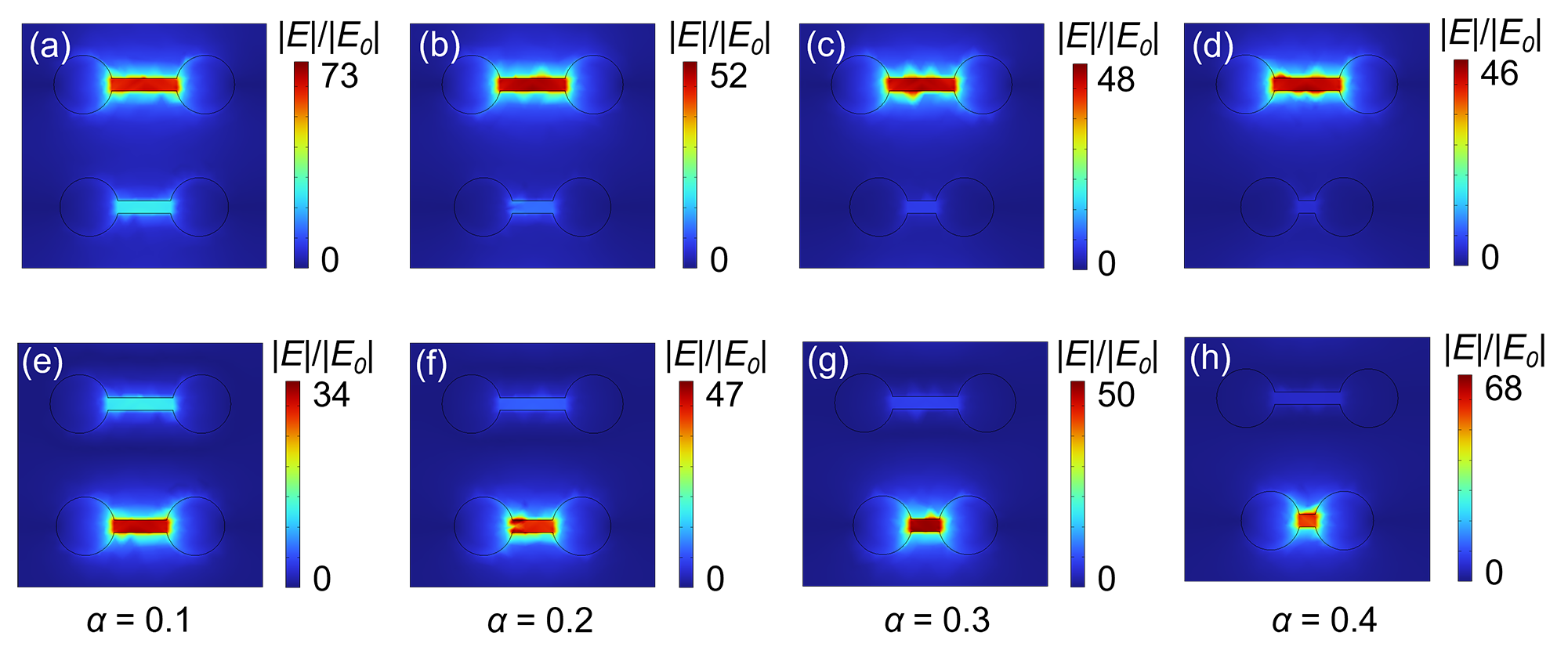


**Figure S4.** Electric field distributions of QBIC (a-d) and anapole mode (e-h) for different *α*.

The evolution of near-field electric distributions for QBIC and anapole mode are shown in Figure S4. The hot spots of QBIC and anapole mode locate within the gaps of D1and D2, respectively. With increasing *α*, the electric fields of QBIC mode gradually decrease whereas the electric fields for anapole exhibits a significant enhancement. For the structure of *α* = 0.4, the field enhancement of anapole mode is stronger than that of QBIC mode while the hot spot of QBIC is much broader.
